# Prognostic and predictive value of ultrasound-based estimated ankle brachial pressure index at early follow-up after endovascular revascularization of chronic limb-threatening ischaemia: a prospective, single-centre, service evaluation

**DOI:** 10.1016/j.eclinm.2023.102410

**Published:** 2024-01-05

**Authors:** Alexander D. Rodway, Lydia Hanna, Jenny Harris, Rachael Jarrett, Charlotte Allan, Felipe Pazos Casal, Benjamin C.T. Field, Martin B. Whyte, Nikolaos Ntagiantas, Ivan Walton, Ajay Pankhania, Simon S. Skene, Gary D. Maytham, Christian Heiss

**Affiliations:** a Vascular Medicine Department, Surrey and Sussex Healthcare NHS Trust, Redhill, UK; b Brighton and Sussex University Hospitals NHS Trust, Brighton, UK; c Imperial College London, London, UK; d School of Health Sciences, University of Surrey, Guildford, UK; e Department of Clinical and Experimental Medicine, University of Surrey, Guildford, UK; f St. George's University Hospitals NHS Foundation Trust, London, UK

**Keywords:** Chronic limb-threatening ischaemia, Peripheral artery disease, Angioplasty, Estimated ankle brachial pressure index, Biomarker

## Abstract

**Background:**

Ankle brachial pressure index can be estimated (eABPI) using cuffless ankle Doppler ultrasound. We evaluated the prognostic value of eABPI measured during pre- and post-procedural ultrasound exams to predict the clinical outcome after endovascular revascularisations.

**Methods:**

In this prospective, single-centre, service evaluation, consecutive patients with symptomatic peripheral artery disease undergoing lower limb endovascular revascularisations between July, 26 2018 and January, 13 2022 at Surrey and Sussex Healthcare NHS Trust (Redhill, UK) were analysed. eABPI was determined using the higher acceleration index measured with angle-corrected duplex ultrasound in ankle arteries before and ≤1 month post-procedure. Clinical outcomes (mortality, major amputations, amputation-free survival [AFS], clinically driven target lesion revascularization [cdTLR], major adverse limb events [MALE; cdTLR and major amputation], wound healing) were assessed over 1 year.

**Findings:**

Of 246 patients treated, for 219 patients (median 75 [IQR 66–83] years) pre- and post-procedural eABPI (0.50 [0.33–0.59] and 0.90 [0.69–1.0], *p* < 0.0001) were available, respectively. In n = 199 patients with chronic limb-threatening ischaemia (CLTI) Kaplan–Meier survival analyses showed that higher post-procedural, but not pre-procedural, eABPI was associated with favourable AFS, MALE, cdTLR, and wound healing. This was confirmed in Cox regression analysis and remained significant with adjustment for pre-procedural eABPI, age, sex, co-morbidities, treated levels, wound score, and foot infection. Whereas all clinical outcomes, except for survival, were significantly better at ≥0.7 *vs* <0.7, wound healing (unadjusted: HR 1.7 (95% CI 1.2–2.6), adjusted: HR 2.1 (95% CI 1.3–3.1), cdTLR, and MALE (unadjusted: HR 0.41 (95% CI 0.18–0.93), adjusted: HR 0.28 (95% CI 0.11–0.74) were significantly improved at ≥0.9 *vs* <0.9.

**Interpretation:**

Post-procedural eABPI can provide valid, clinically important prognostic and predictive information. Our data indicate that revascularisations should target values of at least 0.9 to achieve optimal outcomes. Future studies need to confirm generalisability and cost-effectiveness in a wider context.

**Funding:**

European Partnership on Metrology, co-financed from European Union's Horizon Europe Research and Innovation Programme and 10.13039/100014013UK Research and Innovation.

**Research in context**

**Evidence before this study**

We searched PubMed for studies published until August 01, 2023, using search terms ankle brachial (pressure), estimation, ultrasound, follow-up, surveillance and angioplasty. We also searched references listed in the identified papers. Only one of the studies, our previous publication, demonstrated that ankle brachial pressure index (ABPI) can be estimated and diagnose peripheral artery disease (PAD) with duplex ultrasound (sensitivity: 97%, specificity: 96–100%) as compared to ultrasound and CTA imaging standards independent of diabetes. However, several studies indicate that tibial and pedal waveform analysis (not using values for ABPI) can accurately (sensitivity 83%, specificity: 87%) diagnose PAD better than standard ABPI (sensitivity 63%, specificity: 89%) and toe brachial index (sensitivity 83%, specificity: 66%). Whereas previous studies have indicated that ultrasound surveillance can be useful after angioplasty to assess patency and that surveillance can be useful after angioplasty, no study has evaluated if and how post-procedural ABPI values can predict the outcome after angioplasty.

**Added value of this study**

To our knowledge, this is the first study to show that post-procedural eABPI can predict the 1-year clinical outcome after angioplasty in patients with chronic limb-threatening ischaemia (CLTI). It also provides preliminary evidence that a value of 0.9 should be the target for optimal results of revascularisations.

**Implications of all the available evidence**

Current clinical practise guidelines for PAD and CLTI have started to cautiously recommend post-procedural follow-up exams, but there is a lack of evidence to support recommendations how this should be done (clinical exam, duplex ultrasound or ABPI) and what the target criteria may be. With a wider view, early diagnosis and treatment of PAD have been a problem due to the limitations related to the validity and availability of the standard ABPI method for many patients. The current study takes the concept of eABPI measurements one step further along the development pathway. Due to the simplicity and broad applicability of the method, eABPI could become an integral part of vascular ultrasound exams adding significant value and capability also outside of vascular medicine and surgery i.e., emergency departments, radiology, and cardiology.

## Introduction

Chronic limb-threatening ischaemia (CLTI) is an advanced stage of peripheral artery disease (PAD) with lower limb rest pain, gangrene, and/or ulceration.^1^ Patients affected by CLTI are at extremely high risk of mortality and amputation. Revascularisation plays a major role in the treatment of CLTI as it can promote wound healing and improve amputation-free survival.^2^ Whereas the ‘success’ of revascularisation appears inherently critical to achieve optimal outcomes, it is often challenging to quantify, for both clinicians and patients.

The ankle brachial pressure index (ABPI) is the first-line test to diagnose PAD and assess the severity.^3^, ^4^, ^5^ However, it has not been adopted widely in healthcare systems.^6^^,^^7^ In high risk patient groups such as diabetes and renal failure, media sclerosis may result in both false positive and false negative results^8^ - missing up to 39% of PAD in patients (sensitivity 61%).^9^^,^^10^ Furthermore, ABPI measurement is time-consuming, uncomfortable for patients, and heavily operator-dependent.^6^ In patients with diagnosed PAD and in particular CLTI having undergone revascularisations, non-invasive testing with ABPI is a recommended part of surveillance programs to provide an objective assessment of the success of PAD interventions, detect restenosis and monitor disease progression.^5^^,^^11^^,^^12^ There are no published studies that have directly assessed the value of post-procedural ABPI measurements in this context, i.e., directly demonstrated whether post-angioplasty ABPI can predict the clinical outcome and the optimal value to prevent adverse outcomes. Due to the lack of evidence, recommendations for ABPI measurement and surveillance after endovascular revascularisations in clinical guidelines are un-graded and classified as ‘good practice statements’.^5^

We have recently demonstrated that ABPI can be estimated more accurately than standard compression ABPI with ultrasound, based on the acceleration index in the arteries at the ankle (eABPI).^13^ The performance was excellent, achieving a receiver operating characteristic (ROC) ≥0.98, sensitivity of 97% and specificity 96–100% (using an eABPI cut-off of ≤0.9). This included patients with CLTI, diabetes, and media sclerosis, using vascular duplex ultrasound and computed tomography angiography as imaging reference standards. Its utility in post-procedural assessment of patients has not been investigated.

Whether eABPI can provide important prognostic information in the context of surveillance after endovascular revascularisations is not clear. Here, we evaluate the prognostic value of eABPI measured during pre- and post-procedural ultrasound exams to predict the clinical outcome after endovascular revascularisations.

## Methods

### Study design and participants

A single-centre all-comers prospective service evaluation was performed including consecutive patients undergoing lower limb endovascular revascularisation from July 2018 to January 2022 at East Surrey Hospital, Redhill, UK. Data were prospectively collected at the time of the procedure and during routine care of the patients. Earlier data from this programme have been published previously.^14^ The hospital is a secondary care district general hospital supported by the vascular hubs at Brighton and Sussex Medical School and St. George's University Hospital NHS Foundation Trust in London with vascular surgeons and interventional radiologists on joint appointments. Clinical outcomes (mortality, major amputations, amputation-free survival [AFS], clinically driven target lesion revascularization [cdTLR], major adverse limb events [MALE; cdTLR, major amputation], wound healing) were assessed over the first year after endovascular revascularisation based on electronic healthcare records. We adhered to *The Strengthening the Reporting of Observational Studies in Epidemiology (STROBE)* guidelines.

The study was conducted in accordance with the Declaration of Helsinki, and all measurements were conducted as part of routine clinical care within a National Health Service acute hospital in the UK. The confidentiality of patient data was strictly observed, and only anonymised data were analysed. As a service evaluation, there was no requirement for research ethics committee approval. Patient consent was waived as this was part of a service evaluation without the requirement for research ethics committee approval.

All patients were presented and discussed at a weekly multi-disciplinary team (vascular surgery, angiology, interventional radiology in attendance) or multi-disciplinary diabetic foot team (vascular surgery, angiology, diabetology, microbiology, podiatry, tissue viability in attendance) meetings. The diagnosis of PAD was based on clinical assessment, eABPI ≤0.9 with concordant Doppler waveforms, and/or imaging, and the classification into clinical stages according to Fontaine: Fontaine II for claudication without rest pain or tissue loss and Fontaine III or IV when rest pain or ischaemic ulcers/gangrene were present and plausibly related to ischaemia. Chronic limb-threatening ischaemia (CLTI) was defined as Fontaine III and IV. Indications for revascularisation were symptomatic PAD based on current PAD treatment guidelines,^3^^,^^5^ i.e., short distance (life-style limiting) claudication not responding to or amenable to exercise therapy, rest pain, non-healing ulcer or gangrene. Individualized optimal revascularization modality (angioplasty *vs* open surgery), surgical risk, technical feasibility and procedure planning were assessed by the team's interventionalists and vascular surgeons based on clinical characteristics, symptoms and imaging including duplex ultrasound and CT angiogram.

### Procedures

All procedures were performed by an experienced consultant vascular physician or interventional radiologist, following contemporary treatment guidelines, with the objective of achieving at least a single vessel straight line flow to the foot. In case of crural artery disease, preference was given to the artery supplying the angiosome in which the ulcer or gangrene was present. All patients received a duplex ultrasound scan of the access sites, on table, by the interventionalist. Punctures were mostly performed under ultrasound guidance aiming at a common femoral artery. Technical success was defined as the visually successful treatment of the target lesion(s) with less than 30% remaining stenosis.

Post-procedure, unless there was a contra-indication, patients were started on dual antiplatelet therapy consisting of daily Aspirin 75 mg and Clopidogrel 75 mg for 1–3 months with a Clopidogrel loading dose of 300 mg. In case of oral anti-coagulation, only clopidogrel was added. If possible, vascular occlusion devices were used (Angioseal, Terumo, USA). Patients were allowed a small breakfast up to 3 h before the procedure. Afterwards, patients lay flat for 1 h followed by 1 h of bed rest with elevated head, followed by a light snack and drink if desired, and discharge after groin check at 4 h. If access was difficult or there was superficial bleeding visible through the transparent dressing, a duplex ultrasound was performed to exclude haematoma or pseudo-aneurysm. All patients were scheduled within a month of discharge for a duplex ultrasound examination of the access site and intervened vessel segment together with eABPI and vascular clinic review. Wound care continued throughout within our network including local podiatry.

As described previously,^13^ eABPI was calculated based on the higher acceleration index (AccI) measured with angle-corrected duplex ultrasound at the ankle (anterior or posterior tibial artery) (eABPI = 0.297 ∗ ln[0.039 ∗ AccI + 1]). An Excel spreadsheet for simple calculation is provided as a supplement to the original publication.^13^ The AccI was determined automatically by the onboard software of the ultrasound machine (GE Vivid S9 and Samsung) and represents the slope in cm/s^2^ of the connecting line between the foot and the peak of the systolic upstroke of the arterial Doppler curve envelope.

### Statistical analyses

Most data were not normally distributed (Kolmogorov–Smirnov test *p* < 0.05). Data are presented as median and interquartile range. Between group comparisons were performed with Mann Whitney U test when 2 groups were compared and Kruskal–Wallis test of >2 groups were compared. Wound closure, mortality, major amputation, or target lesion revascularization (cdTLR) within 1 year following angioplasty were assessed with Kaplan–Meier survival analysis and Log Rank (Mantel–Cox) test according to qualitative change in eABPI (increased, unchanged/decreased), quartiles of post-procedural eABPI and different eABPI cut-offs (<0.7 *vs* ≥0.7, <0.8 *vs* ≥0.8, <0.9 *vs* ≥0.9) in patients with CLTI. Sensitivity analyses included Kaplan–Meier analyses of 1 year outcome in all patients according to PAD status (claudication/CLTI and Fontaine stages), baseline and post-procedural eABPI quartiles and in patients with CLTI, by missing post-procedural eABPI, and technical success. We performed Cox regression analyses to allow adjustment for confounding variables. We compared four models to examine the association between post-procedural eABPI and clinical outcomes. Model 1 treated post-procedural eABPI as a continuous variable. Models 2–4 treated eABPI as a categorical variable with cut-offs at <0.7, <0.8, and <0.9, respectively. All models were adjusted for baseline eABPI, age, sex, number of co-morbidities, number of intervened segments (iliac, femoral, popliteal, crural), WIfI wound score (0–3),^5^^,^^15^ and WIfI foot infection score (0–3).^5^^,^^15^ The number of co-morbidities included atrial fibrillation, diabetes mellitus, coronary artery disease, chronic lung disease, arterial hypertension, chronic kidney disease, history of cancer or stroke. We checked the assumption of proportionality by plotting the complementary log–log function [log(minus log)] *vs* time for ‘groups’ within the data to ensure the resulting curves were parallel. The linearity assumption was checked by plotting partial residuals of covariates *vs* time. Analyses were performed with SPSS 29 (IBM).

### Role of the funding source

The funder of the study had no role in study design, data collection, data analysis, data interpretation, or writing of the report. CH, SSS, JH, LH, ADR had access to the dataset and final responsibility for the decision to submit for publication.

## Results

### Baseline characteristics

In *n* = 246 patients (age: 75 [66–83] years) undergoing endovascular revascularisation, pre-procedural eABPIs were available. In *n* = 219 both pre- and post-procedural eABPIs were available (pre: 0.50 [0.33–0.59], post: 0.90 [0.69–1.0], *p* < 0.0001). Most patients were treated for CLTI (92%, *n* = 226) and only 8% (*n* = 20) had short distance claudication (Fontaine IIb). The baseline demographic and procedural characteristics of all patients (*n* = 246) and subgroup of patients with CLTI are summarised in Tables 1 and 2, and characteristics are shown separately for those with both pre- and post-procedural eABPIs (*n* = 219 total, *n* = 199 with CLTI).

**Table 1:** Clinical and demographic characteristics of patient populations.

	CI&CLTI	CI&CLTI	CLTI	CLTI	Q1	Q2	Q3	Q3	*p* (Kruskal Wallis U)
	Total (with pre-procedural eABPI)	Total (with pre- and post–procedural eABPI)	Total (with pre-procedural eABPI)	Total (with pre- and post–procedural eABPI)					
*n*	246	219	226	199	55	44	44	56	
Pre-procedural eABPI, median (IQR)	0.50 (0.33–0.59)	0.50 (0.33–0.59)	0.50 (0.33–0.59)	0.50 (0.33–0.59)	0.4 (0.26–0.50)	0.50 (0.31–0.63)	0.58 (0.37–0.69)	0.50 (0.41–0.69)	<0.0001
Post-angioplasty eABPI, median (IQR)		0.90 (0.69–1.0)		0.87 (0.69–1.0)	0.59 (0.41–0.69)	0.79 (0.79–0.83)	0.90 (0.90–0.95)	1.0 (1.0–1.1)	<0.0001
Sex (m/f), *n*	157/89	137/82	141/85	121/78	35/20	26/18	30/14	30/26	
Outpatients/inpatients, *n*	170/76	155/64	150/76	135/64	36/19	36/8	22/22	41/15	
Age, median (IQR), years	75 (66–83)	73 (66–83)	76 (67–84)	76 (66–83)	79 (70–85)	74 (64–81)	76 (67–85)	72 (65–80)	0.12
Haemoglobin, median (IQR), g/l	124 (109–138)	124 (108–136)	123 (108–136)	122 (108–136)	121 (103–135)	127 (115–140)	120 (107–129)	123 (107–138)	0.37
Leucocytes, median (IQR), ×10^9^/l	8.6 (7.3–10.5)	8.6 (7.3–10.3)	8.8 (7.3–10.7)	8.7 (7.4–10.6)	8.3 (7–103)	8.6 (7.5–11.4)	8.7 (7.1–10.9)	8.9 (7.6–10.8)	0.66
Platelets, median (IQR), ×10^9^/l	274 (224–362)	276 (225–355)	281 (228–372)	281 (231–366)	280 (222–322)	271 (234–338)	292 (219–392)	300 (238–391)	0.37
Estimated glomerular filtration rate, median (IQR), ml/min	61 (45–78)	61 (45–78)	60 (45–77)	61 (45–78)	61 (41–79)	64 (38–82)	62 (45–78)	58 (48–78)	0.98
Total cholesterol, median (IQR), mmol/l	4.1 (3.3–5.3)	4.1 (3.4–5.3)	4.0 (3.3–5.3)	4.1 (3.3–5.3)	3.8 (3.4–5.0)	4.0 (3.2–4.9)	4.5 (3.7–5.4)	4.4 (3.1–5.4)	0.36
Low–density lipoprotein cholesterol, median (IQR), mmol/l	1.9 (1.4–3.0)	1.9 (1.4–2.9)	2.0 (1.4–3.0)	2.0 (1.4–3.0)	1.8 (1.3–2.9)	1.7 (1.3–2.9)	2.5 (1.6–3.0)	2.1 (1.2–3.1)	0.51
High–density lipoprotein cholesterol, median (IQR), mmol/l	1.3 (1.0–1.6)	1.3 (1.0–1.6)	1.3 (1.0–1.6)	1.3 (1.0–1.6)	1.4 (1.1–1.7)	1.3 (1.0–1.5)	1.3 (1.0–1.6)	1.2 (1.0–1.5)	0.090
Triglycerides, median (IQR), mmol/l	1.5 (1.1–2.1)	1.5 (1.1–2.2)	1.5 (1.1–2.1)	1.5 (1.1–2.1)	1.3 (1.0–1.7)	1.5 (1.1–2.3)	1.4 (1.1–2.5)	1.7 (1.3–2.4)	0.017
International Normalized Ratio, median (IQR)	1.1 (1.0–1.2)	1.1 (1.0–1.2)	1.1 (1.0–1.2)	1.1 (1.0–1.2)	1.1 (1.0–1.1)	1.0 (1.0–1.1)	1.1 (1.0–1.3)	1.1 (1.0–1.1)	0.10
HbA1c, median (IQR), mmol/mol	49 (41–61)	50 (41–62)	50 (41–63)	50 (41–65)	45 (41–52)	52 (38–69)	45 (40–65)	54 (48–71)	0.094
Systolic blood pressure, median (IQR), mmHg	146 (135–162)	145 (135–160)	146 (135–165)	145 (135–165)	150 (136–165)	143 (133–160)	146 (131–161)	146 (138–166)	0.74
Diastolic blood pressure, median (IQR), mmHg	83 (75–89)	82 (75–89)	83 (75–90)	81 (74–90)	85 (77–90)	80 (70–89)	79 (69–90)	80 (75–89)	0.28
Fontaine (IIb/III/IV), *n*	20/27/199	20/25/174	0/27/199	0/25/174	0/6/49	0/5/39	0/4/40	0/10/46	0.55
Chronic limb–threatening ischaemia, *n*	226	199	226	199	55	44	44	56	>0.99
Rest pain, *n*	181	159	181	159	48	32	34	45	0.33
Ulcer/tissue loss, *n*	199	174	199	174	49	39	40	46	0.55
Ulcer time, median (IQR), d				94 (40–181)	118 (53–198)	64 (40–134)	100 (39–286)	98 (26–174)	0.49
Ulcer time unknown, *n*				67	17	13	16	21	
WIfI wound score (0/1/2/3), *n*				0/31/107/36	0/11/27/11	0/9/28/2	0/5/22/13	0/6/30/10	0.046
WIfI ischaemia score (0/1/2/3), *n*				7/35/78/54	0/1/26/22/0	1/8/19/11	1/14/15/10	5/12/18/11	<0.0001
WIfI foot infection score (0/1/2/3), *n*				56/77/41/0	13/20/16/0	14/20/5/0	16/17/7/0	13/20/13/0	0.16
WIfI stage (0/1/2/3), *n*				1/10/57/106	0/1/3/9/37	0/4/15/20	0/1/15/24	1/2/18/25	0.10
Diabetes mellitus, *n*	147	132	145	126	29	25	30	42	0.17
Diabetes type 1/type 2, *n*	15/126	12/115	15/124	12/113	4/24	2/23	1/27	5/36	0.19
Chronic kidney disease, n	130	112	123	105	28	21	25	31	0.95
Arterial hypertension, *n*	207	183	192	164	42	37	38	47	0.65
Coronary artery disease, *n*	102	86	92	76	23	20	13	20	0.31
Chronic heart failure, *n*	45	34	43	31	13	9	5	4	0.021
Stroke, *n*	30	24	28	20	5	4	5	6	0.93
Cancer, *n*	29	27	26	23	10	3	5	5	0.16
Chronic lung disease, *n*	61	54	53	46	11	10	13	12	0.90
Atrial fibrillation, *n*	60	48	57	44	14	9	9	12	0.82
Smoker/ex-smoker, *n*	47/175	45/152	44/158	41/135	10/32	10/31	5/36	16/33	0.03
Oral anticoagulation, *n*	68	54	65	51	14	9	13	15	0.83
Metformin, *n*	85	75	83	70	15	14	14	27	0.21
Insulin, *n*	69	62	69	61	11	14	14	22	0.25
Aspirin, *n*	98	89	90	78	20	22	15	21	0.60
Clopidogrel, *n*	98	90	88	79	22	23	15	19	0.34
Rivaroxaban 2.5 mg BID, *n*	10	9	9	8	3	1	2	2	0.69
Statin, *n*	160	143	145	126	33	33	25	35	0.11
Ezetimibe, *n*	1	1	1	1	0	0	0	1	0.47
PCSK9-inhibitor, *n*	1	1	1	1	0	0	0	1	0.47
ACEI/ARB, *n*	146	129	134	114	29	23	28	34	0.57
Beta blocker, *n*	92	79	84	71	15	14	16	26	0.27
Diuretic, *n*	4	4	4	4	1	1	1	1	0.99
Calcium channel blocker, *n*	58	55	54	49	16	13	8	12	0.49

ACEI/ARB = Angiotensin–converting enzyme inhibitor/angiotensin receptor blocker, BID = twice daily, eABPI = estimated ankle brachial pressure index, IQR = interquartile range, PCSK9 = proprotein convertase subtilisin/kexin type 9.

**Table 2:** Procedural characteristics.

	CI&CLTI	CI&CLTI	CLTI	CLTI	Q1	Q2	Q3	Q3	*p* (Kruskal Wallis)
	Total (with pre-procedural eABPI)	Total (with pre- and post–procedural eABPI)	Total (with pre-procedural eABPI)	Total (with pre- and post–procedural eABPI)					
*n*	246	219	226	199	55	44	44	56	
Iliac, *n*	40	39	27	26	10	4	7	5	0.35
Femoral, *n*	145	131	137	123	36	31	28	28	0.15
Popliteal, *n*	88	80	86	78	27	16	16	19	0.41
Crural, *n*	125	110	125	110	25	27	26	32	0.37
Number treated levels (1/2/3/4/5), *n*	117/81/39/2/1	104/73/36/2/1	104/77/38/2/1	91/69/35/2/1	22/20/12/1/0	17/19/8/0/0	18/16/8/0/1	34/14/7/1	0.12
Side (R/L/bilateral), *n*	107/128/11	96/113/9	101/117/7	90/102/6	28/25/2	14/28/2	23/20/0	25/29/2	
Recanalisation, *n*	203	182	190	169	43	37	36	53	0.093
Total lesion length, mean (SD), mm	140 (75–265)	157 (80–300)	157 (80–287)	190 (85–300)	150 (50–250)	160 (65–290)	220 (107–312)	200 (87–300)	0.31
Stenting, *n* patients	135	128	120	113	31	24	30	28	0.25
Bare metal stents, *n* patients	74	69	60	55	20	9	15	11	0.33
Drug eluting stents, *n* patients	61	59	60	58	11	15	15	17	0.33
Total stent length, median (IQR), mm	80 (40–145)	80 (40–142)	80 (40–152)	80 (40–150)	100 (39–150)	140 (40–179)	80 (40–130)	68 (39–130)	0.79
Drug coated balloons, *n* patients	84	82	80	78	19	20	22	17	0.34
Total length drug-coated balloon, median (IQR), mm	160 (80–240)	160 (80–240)	160 (80–240)	160 (80–240)	160 (120–240)	200 (120–240)	220 (85–295)	120 (38–240)	0.56
Fluoroscopy time, median (IQR), s	1141 (666–1579)	1127 (663–1515)	1160 (681–1620)	1145 (680–1551)	1131 (458–1444)	1140 (605–1522)	1109 (708–1458)	1288 (857–2059)	0.13
Contrast volume, median (IQR), ml	80 (74–110)	85 (65–115)	85 (65–116)	90 (70–120)	80 (60–120)	90 (62–124)	80 (70–105)	90 (70–120)	0.94
Technical success, *n*	224	208	206	190	49	43	44	54	0.049
Minor procedure related complication, *n*	12	11	10	9	2	4	1	2	0.42
Major complication, *n*	0	0	0	0	0	0	0	0	
Time to procedure, median (IQR), d	8 (6–21)	8 (6–22)	8 (6–17)	8 (6–17)	10 (6–22)	14 (7–21)	8 (6–15)	8 (1–15)	0.41

**Fig. 1:**
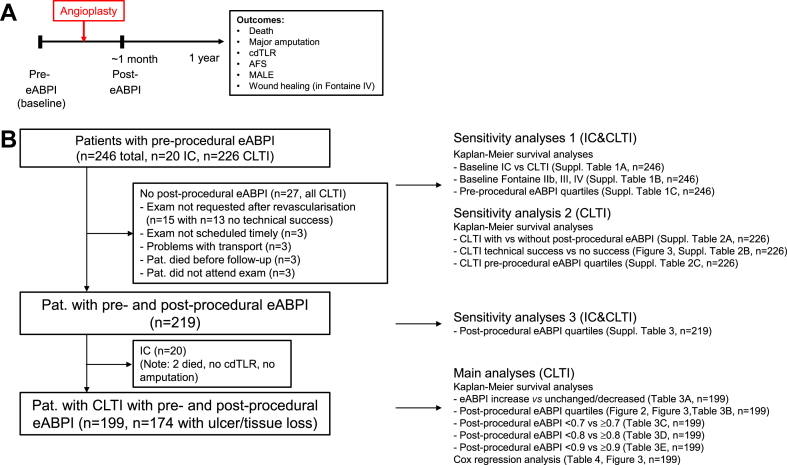
Overview of (A) study design and (B) flowchart of patient groups and analyses (AFS = amputation-free survival, IC = intermittent claudication, CLTI = chronic limb-threatening ischaemia, cdTLR = clinically driven target lesion revascularisation, MALE = major adverse limb event [cdTLR, major amputation]).

We first explored the reasons for missing post-procedural eABPI. All patients with missing post-procedural eABPI (*n* = 27, Fig. 1) had CLTI. The most frequent reason for missing data was that the exam was not requested after revascularisation by the interventional team (*n* = 15) or not scheduled by ultrasound team (*n* = 3). In most of the cases (*n* = 13), when exams were not requested by interventional team, the revascularisation procedure was classified as ‘not technically successful’. In these patients, the interventional team deemed repeat eABPI measurement unnecessary as the vascular status at the end of the procedure appeared angiographically and clinically unchanged. In another 3 patients, transport could not be organised, 3 died before scheduled exam and 3 did not attend the appointment - with no reason given. Twenty-one of the patients attended clinical follow-up appointments. Of the six that did not, 3 had died and no reasons were provided for the remaining 3 patients.

Over the 1-year follow-up of the 246 patients, 52 patients died, 16 had major amputations, and 15 required cdTLR (Supplementary Table 1). Almost all events occurred in patients with CLTI. In patients with claudication, only 2 patients died (unrelated to limb) and no amputations or cdTLR were required. In the 196 patients with Fontaine IV, 113 wounds completely healed.

Due to the small number of patients with claudication and the low event rates in this group, the main analyses were based on the 199 patients with CLTI. Additional sensitivity analyses conducted in the entire cohort and taking the availability of post-procedural eABPI and technical success into account are provided as Supplementary Table 2.

### Clinical outcome prediction based on post-procedural eABPI improvement and post-procedural eABPI values

We performed Kaplan–Meier (KM) survival analyses in patients with CLTI comparing outcomes of patients with post-procedurally increased eABPI (*n* = 182, 91.5%) with those that were unchanged or decreased (*n* = 17, 8.5%) as compared to pre-procedure eABPI (Table 3A). Patients with increased eABPI had significantly improved 1-year mortality (increased eABPI: 17% *vs* unchanged/decreased: 37%, *p* = 0.029) and AFS (increased: 76% *vs* unchanged/decreased: 56%, *p* = 0.042). In those with chronic wounds or tissue loss (*n* = 174), significantly more wounds healed (increased: 84% *vs* unchanged/decreased: 47%, *p* = 0.020).

**Table 3:** Kaplan Meier survival analysis based on (A) qualitative change of estimated ankle brachial pressure index (eABPI) (increased *vs* unchanged/decreased compared to pre-procedure), (B) quartiles of post-procedural eABPI in patients with chronic limb-threatening ischaemia (CLTI; n = 199, n = 174 with ulcer/gangrene) and comparing post-procedural eABPI (C) < *vs* ≥ 0.7, (D) < *vs* ≥ 0.8 and (E) < *vs* ≥ 0.9.

A. Qualitative post-procedure eABPI change	All (CLTI)	eABPI increased	eABPI unchanged/decreased	*p* (Mann–Whitney U)
Mean post-procedural eABPI–median (IQR)	0.87 (0.69–1.0)	0.90 (0.71–1.0)	0.50 (0.31–0.59)	**<0.0001**

	Total *n*	Events *n*	Mean (95% CI)	Total *n*	Events *n*	Mean (95% CI)	Total *n*	Events *n*	Mean (95% CI)	*p* (log rank Mantel–Cox)
1 year death	199	37 (19%)	319 (305–333)	182	31 (17%)	324 (310–338)	17	6 (37%)	261 (192–329)	0.029
1 year major amputation	199	13 (7%)	349 (340–358)	182	12 (7%)	350 (341–359)	17	1 (7%)	342 (341–359)	0.89
1 year cdTLR	199	15 (8%)	341 (330–353)	182	13 (8%)	344 (332–355)	17	2 (13%)	322 (266–378)	0.37
1 year major amputation or death	199	50 (26%)	304 (289–320)	182	43 (24%)	310 (294–326)	17	7 (44%)	241 (170–313)	0.042
1 year MALE	199	25 (14%)	329 (315–343)	182	23 (14%)	328 (314–342)	17	3 (19%)	300 (235–366)	0.34
1 year wound healing	174	108 (81%)	193 (173–213)	159	103 (84%)	185 (165–206)	15	5 (47%)	283 (211–355)	0.020
			Median: 168 (129–207)			Median: 162 (129–195)				

B. Post-procedure eABPI quartiles (range)	Q1 (0–0.69)	Q2 (0.70–0.86)	Q3 (0.87–0.99)	Q4 (1.0–1.3)	*p* (Kruskal–Wallis)
Post-procedural eABPI–median (IQR)	0.59 (0.0–0.69)	0.79 (0.79–0.83)	0.90 (0.90–0.95)	1.0 (1.0–1.1)	<0.0001

	Total *n*	Events *n*	Mean (95% CI)	Total *n*	Events *n*	Mean (95% CI)	Total *n*	Events *n*	Mean (95% CI)	Total *n*	Events *n*	Mean (95% CI)	*p* (log rank Mantel–Cox)
1 year death	55	15 (28%)	289 (256–323)	44	5 (11%)	341 (319–363)	44	8 (18%)	322 (292–352)	56	9 (16%)	328 (304–351)	0.14
1 year major amputation	55	7 (15%)	323 (293–352)	44	1 (2%)	359 (348–370)	44	2 (5%)	358 (349–367)	56	3 (6%)	358 (347–369)	0.055
1 year cdTLR	55	8 (16%)	311 (177–345)	44	3 (7%)	348 (328–368)	44	0		56	4 (8%)	344 (323–364)	0.034
1 year major amputation or death	55	22 (42%)	251 (214–289)	44	6 (14%)	335 (312–359)	44	10 (23%)	317 (287–347)	56	12 (22%)	321 (295–346)	0.0035
1 year MALE	55	14 (28%)	280 (241–318)	44	4 (9%)	342 (320–365)	44	2 (5%)	358 (349–367)	56	6 (12%)	327 (315–360)	0.0021
1 year wound healing	49	19 (61%)	248 (208–289)	39	24 (73%)	188 (144–232)	40	30 (95%)	169 (130–208)	46	35 (92%)	170 (136–214)	0.0016
			Median: 276 (152–400)			Median: 168 (142–212)			Median: 137 (69–205)			Median: 140 (92–188)	

C. Post-procedure eABPI <0.7/≥0.7	<0.7	≥0.7	*p* (Mann–Whitney U)
mean post-procedural eABPI–median (IQR)	0.59 (0.41–0.69)	0.91 (0.84–1.0)	<0.0001

	Total *n*	Events *n*	Mean (95% CI)	Total *n*	Events *n*	Mean (95% CI)	*p* (log rank Mantel–Cox)
1 year death	55	15 (29%)	289 (256–323)	144	22 (16%)	330 (316–345)	0.028
1 year major amputation	55	7 (15%)	322 (193–352)	144	6 (5%)	358 (352–364)	0.0073
1 year cdTLR	55	8 (16%)	313 (280–346)	144	7 (5%)	351 (341–362)	0.0093
1 year major amputation or death	55	22 (42%)	251 (213–290)	144	28 (20%)	323 (308–339)	0.00038
1 year MALE	55	14 (29%)	280 (241–318)	144	12 (9%)	345 (333–357)	0.00019
1 year wound healing	49	19 (61%)	248 (209–289)	125	89 (87%)	176 (153–198)	0.0020
			Median: 276 (152–400)			Median (146 (120–172)	

D. Post-procedure eABPI <0.8/≥0.8	<0.8	≥0.8	*p* (Mann–Whitney U)
Mean post-procedural eABPI–median (IQR)	0.69 (0.50–0.75)	0.97 (0.90–1.0)	<0.0001

	Total *n*	Events *n*	Mean (95% CI)	Total *n*	Events *n*	Mean (95% CI)	*p* (log rank Mantel–Cox)
1 year death	83	19 (24%)	305 (280–330)	116	18 (16%)	328 (312–345)	0.14
1 year major amputation	83	8 (11%)	335 (315–355)	116	5 (5%)	359 (353–365)	0.080
1 year cdTLR	83	11 (14%)	322 (298–346)	116	4 (4%)	354 (345–365)	0.0063
1 year major amputation or death	83	27 (34%)	277 (249–306)	116	23 (20%)	323 (306–341)	0.019
1 year MALE	83	18 (24%)	297 (269–326)	116	8 (8%)	349 (337–361)	0.00098
1 year wound healing	73	32 (61%)	233 (198–267)	101	76 (94%)	169 (146–193)	0.00046
			Median: 153 (95–411)			Median: 145 (123–167)	

E. Post-procedure eABPI <0.9/≥0.9	<0.9	≥0.9	*p* (Mann–Whitney U)
Mean post-procedural eABPI–median (IQR)	0.69 (0.56–0.79)	1.0 (0.90–1.0)	<0.0001

	Total *n*	Events *n*	Mean (95% CI)	Total *n*	Events *n*	Mean (95% CI)	*p* (log rank Mantel–Cox)
1 year death	101	21 (22%)	310 (288–332)	98	16 (17%)	328 (310–346)	0.34
1 year major amputation	101	8 (9%)	341 (324–357)	98	5 (6%)	358 (350–365)	0.32
1 year cdTLR	101	11 (12%)	330 (310–350)	98	4 (4%)	353 (341–365)	0.055
1 year major amputation or death	101	29 (30%)	288 (263–312)	98	21 (22%)	321 (302–340)	0.15
1 year MALE	101	18 (20%)	310 (286–333)	98	8 (9%)	346 (332–360)	0.028
1 year wound healing	84	44 (67%)	217 (187–247)	90	64 (94%)	171 (145–197)	0.0046
			Median: 207 (130–284)			Median: 143 (115–171)	

cdTLR = clinically driven target lesion revascularisation, MALE = major adverse limb event [cdTLR, major amputation], total *n* are number at risk.

**Fig. 2:**
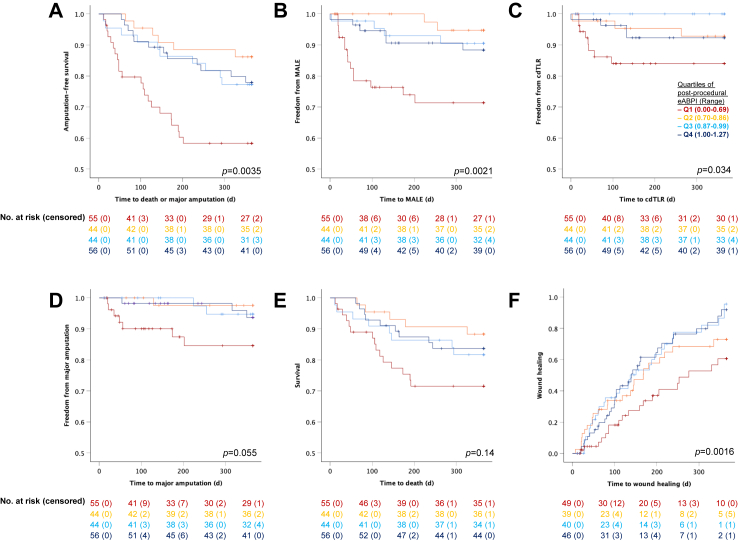
Kaplan-Maier survival analysis for 1-year (A) amputation-free survival, (B) freedom from major adverse limb events (MALE; major amputation and clinically driven target lesion revascularisation [cdTLR]), (C) freedom from cdTLR, (D) freedom from major amputation, (E) overall survival, and (F) wound healing in patients with chronic limb-threatening ischaemia (*n* = 199, *n* = 174 with ulcers/gangrene) (Overall *p*-values are shown, see Table 3 for 95% confidence intervals of individual groups).

We then performed KM survival analyses according to quartiles of post-procedural eABPI in the patients with CLTI (Q1 [Range: 0.00–0.69], Q2 [0.70–0.86], Q3 [0.86–0.99], Q4 [1.00–1.27]; Table 3B, Fig. 2). In those with ulcers (n = 174), wound healing was significantly improved in the groups with higher post-procedural eABPI (Q1: 61%, Q2: 73%, Q3: 95%, Q4: 92%; *p* = 0.0016). In all patients with CLTI (*n* = 199), higher post-procedural eABPI was associated with significantly higher AFS (Q1: 58%, Q2: 86%, Q3: 77%, Q4: 78%; *p* = 0.0035) and lower MALE (Q1: 28%, Q2: 9%, Q3: 5%, Q4: 12%; *p* = 0.0019) and cdTLR (Q1: 16%, Q2: 7%, Q3: 0%, Q4: 8%; *p* = 0.034). Mortality and major amputation were also more favourable in Q2–4 than in Q1 but this did not reach statistical significance when analysing data by quartiles.

**Fig. 3:**
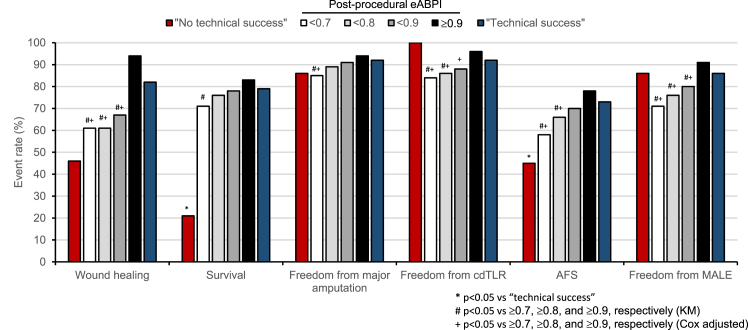
Overview of event rates for endpoints according to technical success and post-procedural eABPI. Based on data detailed in Table 3 and Supplementary Table 2; ∗*p* < 0.05 *vs* “technical success”, #*p* < 0.05 *vs* ≥0.7, ≥0.8, and ≥0.9, respectively (Kaplan–Meier analysis, Table 3), +*p* < 0.05 *vs* ≥0.7, ≥0.8, and ≥0.9, respectively (adjusted Cox regression analysis, Table 4) (AFS = amputation-free survival, MALE = major adverse limb event [cdTLR, major amputation]).

We then compared outcomes between patients with CLTI with post-procedural eABPI values of ≥0.7 (equivalent to Q2–4) with those that had reached values <0.7 (equivalent to Q1). This showed that all outcomes were statistically significantly improved in patients with post-procedural eABPI values of ≥0.7 (Table 3C). Patients with post procedural eABPI ≥0.8 had significantly better wound healing (*p* = 0.00046) and AFS (*p* = 0.019) as well as lower cdTLR (*p* = 0.0063) and MALE (*p* = 0.010) than those <0.8. Patients with post-procedural eABPI ≥0.9 had significantly more wound healing (≥0.9: 94%, <0.9: 67%; *p* = 0.0046) and lower MALE (≥0.9: 9%, <0.9: 20%; *p* = 0.028) than those <0.9. The data confirm the analyses above (by quartiles): that a post-procedural eABPI ≥0.7 is associated with much improved clinical outcome and that values of ≥0.9 still carry significant benefits. The cumulative event rates for all clinical outcomes of patients with post-procedural eABPI <0.7, <0.8, <0.9, and ≥0.9 are shown on Fig. 3 together with KM event rates in patients with procedures categorised based on documented lack or presence of technical success.

Four Cox regression models were used to examine the association between post-procedural eABPI and clinical outcomes adjusted for confounders including baseline eABPI, age, sex, number of co-morbidities, number of intervened segments, wound score, and foot infection score. The analyses confirmed largely the results of the Kaplan–Meier survival analysis (Table 4 and Fig. 3). In Model 1 (eABPI continuous), post-procedural eABPI was a significant independent predictor of freedom from major amputation and MALE as well as AFS and wound healing. Model 2 showed that post-procedural eABPI ≥0.7 independently predicted freedom from major amputation, cdTLR, MALE as well as AFS and more wound healing. In the multivariable adjusted models eABPI was no longer a significant predictor of survival and survival was significantly associated with age and co-morbidities. Importantly, Model 4 confirmed that post-procedural eABPI ≥0.9 was associated with significantly more favourable outcomes in terms of cdTLR, MALE, and wound healing. The Cox models also indicated important roles of factors other than post-procedural eABPI. Interestingly, overall survival was not significantly predicted by post-procedural eABPI in the adjusted models indicating that age, co-morbidities, and foot infection were significant determinants of mortality.

**Table 4:** Cox regression analysis.

A. Death	No at risk *n*	Cummulative death *n* (%)	Hazards ratio (95% CI)	
			Unadjusted	Adjusted for baseline eABPI, age, no. co-morbidities, sex, no. treated levels, wound score, foot infection score
Model 1				
Post-procedural eABPI continuous	199	37 (19%)	0.56 (0.14–2.17)	1.07 (0.24–4.81)
				age: **1.04 (1.01–1.08)**
				co-morbidities: **1.27 (1.03–1.57)**
				foot infection: **2.40 (1.44–3.98)**
Model 2				
Post-procedural eABPI <0.7 (reference)	55	15 (29%)	1	1
Post-procedural eABPI ≥0.7	144	22 (16%)	**0.49 (0.25–0.94)**	0.65 (0.32–1.32)
				age: **1.04 (1.01–1.08)**
				co-morbidities: **1.27 (1.03–1.57)**
				foot infection: **2.36 (1.41–3.95)**
Model 3				
Post-procedural eABPI <0.8 (reference)	83	19 (24%)	1	1
Post-procedural eABPI ≥0.8	116	18 (16%)	0.61 (0.32–1.17)	0.68 (0.34–1.36)
				age: **1.04 (1.00–1.08)**
				co-morbidities: **1.28 (1.03–1.58)**
				foot infection: **2.42 (1.44–4.07)**
Model 4				
Post-procedural eABPI <0.9 (reference)	101	21 (22%)	1	1
Post-procedural eABPI ≥0.9	98	16 (17%)	0.73 (0.38–1.39)	0.72 (0.36–1.46)
				age: **1.04 (1.00–1.08)**
				co-morbidities: **1.28 (1.04–1.58)**
				foot infection: **2.42 (1.44–4.07)**

B. Major amputation	No at risk *n*	Cummulative major amputation *n* (%)	Hazards ratio (95% CI)	
			Unadjusted	Adjusted for baseline eABPI, age, no. co-morbidities, sex, no. treated levels, wound score, foot infection score
Model 1				
Post-procedural eABPI continuous	199	13 (7%)	**0.11 (0.15–0.78)**	0.15 (0.01–1.59)
				age: **0.94 (0.89–0.99)**
Model 2				
Post-procedural eABPI <0.7 (reference)	55	7 (15%)	1	1
Post-procedural eABPI ≥0.7	144	6 (5%)	**0.25 (0.08–0.75)**	**0.20 (0.06–0.73)**
				age: **0.94 (0.89–0.99)**
Model 3				
Post-procedural eABPI <0.8 (reference)	83	8 (11%)	1	1
Post-procedural eABPI ≥0.8	116	5 (5%)	0.38 (0.12–1.17)	0.30 (0.08–1.04)
				age: **0.94 (0.89–0.99)**
Model 4				
Post-procedural eABPI <0.9 (reference)	101	8 (9%)	1	1
Post-procedural eABPI ≥0.9	98	5 (6%)	0.57 (0.19–1.75)	0.46 (0.13–1.61)
				age: **0.94 (0.89–0.99)**

C. Clinically driven target lesion revascularisation	No at risk *n*	Cummulative cdTLR *n* (%)	Hazards ratio (95% CI)	
			Unadjusted	Adjusted for baseline eABPI, age, no. co-morbidities, sex, no. treated levels, wound score, foot infection score
Model 1				
Post-procedural eABPI continuous	199	15 (8%)	0.17 (0.02–1.16)	**0.03 (0.00–0.38)**
Model 2				
Post-procedural eABPI <0.7 (reference)	55	8 (16%)	1	1
Post-procedural eABPI ≥0.7	144	7 (5%)	**0.28 (0.10–0.78)**	**0.07 (0.02–0.31)**
				age: **0.95 (0.91–0.97)**
				baseline eABPI: **56.48 (1.28–2484.70)**
Model 3				
Post-procedural eABPI <0.8 (reference)	83	11 (14%)	1	1
Post-procedural eABPI ≥0.8	116	4 (4%)	**0.23 (0.07–0.73)**	**0.14 (0.04–0.55)**
Model 4				
Post-procedural eABPI <0.9 (reference)	101	11 (12%)	1	1
Post-procedural eABPI ≥0.9	98	4 (4%)	0.34 (0.11–1.08)	**0.22 (0.06–0.84)**

D. Amputation-free survival	No at risk *n*	Cummulative major amputations or death *n* (%)	Hazards ratio (95% CI)	
			Unadjusted	Adjusted for baseline eABPI, age, no. co-morbidities, sex, no. treated levels, wound score, foot infection score
Model 1				
Post-procedural eABPI continuous	199	50 (26%)	**0.29 (0.09–0.91)**	0.43 (0.13–1.47)
				co-morbidities: **1.31 (1.08–1.59)**
				foot infection: **1.75 (1.15–2.66)**
Model 2				
Post-procedural eABPI <0.7 (reference)	55	22 (42%)	1	1
Post-procedural eABPI ≥0.7	144	28 (20%)	**0.38 (0.21–0.66)**	**0.42 (0.23–0.77)**
				co-morbidities: **1.31 (1.08–1.58)**
				foot infection: **1.69 (1.11–2.58)**
Model 3				
Post-procedural eABPI <0.8 (Reference)	83	27 (34%)	1	1
Post-procedural eABPI ≥0.8	116	23 (20%)	**0.52 (0.30–0.91)**	**0.52 (0.29–0.93)**
				co-morbidities: **1.31 (1.08–1.58)**
				foot infection: **1.77 (1.16–2.70)**
Model 4				
Post-procedural eABPI <0.9 (Reference)	101	29 (30%)	1	1
Post-procedural eABPI ≥0.9	98	21 (22%)	0.66 (0.38–1.16)	0.63 (0.34–1.15)
				co-morbidities: **1.31 (1.08–1.59)**
				foot infection: **1.78 (1.17–2.71)**

E. Major adverse limb events	No at risk *n*	Cummulative MALE *n* (%)	Hazards ratio (95% CI)	
			Unadjusted	Adjusted for baseline eABPI, age, no. co-morbidities, sex, no. treated levels, wound score, foot infection score
Model 1				
Post-procedural eABPI continuous	199	25 (14%)	**0.15 (0.03–0.62)**	**0.06 (0.01–0.33)**
				age: **0.96 (0.93–0.99)**
				co-morbidities: **1.46 (1.08–1.98)**
Model 2				
Post-procedural eABPI <0.7 (reference)	55	14 (29%)	1	1
Post-procedural eABPI ≥0.7	144	12 (9%)	**0.25 (0.12–0.55)**	**0.10 (0.04–0.28)**
				age: **0.95 (0.92–0.98)**
				co-morbidities: **1.49 (1.10–2.02)**
Model 3				
Post-procedural eABPI <0.8 (reference)	83	18 (24%)	1	1
Post-procedural eABPI ≥0.8	116	8 (8%)	**0.27 (0.12–0.62)**	**0.18 (0.07–0.47)**
				age: **0.96 (0.92–0.99)**
				co-morbidities: **1.38 (1.03–1.86)**
Model 4				
Post-procedural eABPI <0.9 (reference)	101	18 (20%)	1	1
Post-procedural eABPI ≥0.9	98	8 (9%)	**0.41 (0.18–0.93)**	**0.28 (0.11–0.74)**
				age: **0.96 [0.92–0.99)**
				co-morbidities: **1.44 (1.07–1.95)**

F. Wound healing	No at risk *n*	Cummulative wounds healed *n* (%)	Hazards ratio (95% CI)	
			Unadjusted	Adjusted for baseline eABPI, age, no. co-morbidities, sex, no. treated levels, wound score, foot infection score
Model 1				
Post-procedural eABPI continuous	174	108 (81%)	**3.9 (1.5–10.1)**	**4.28 (1.49–12.29)**
				male sex: **0.55 (0.36–0.85)**
Model 2				
Post-procedural eABPI <0.7 (reference)	49	19 (61%)	1	1
Post-procedural eABPI ≥0.7	125	89 (87%)	**2.1 (1.3–3.5)**	**2.41 (1.40–4.15)**
				male sex: **0.54 (0.35–0.83)**
Model 3				
Post-procedural eABPI <0.8 (reference)	73	32 (61%)	1	1
Post-procedural eABPI ≥0.8	101	76 (94%)	**2.1 (1.4–3.2)**	**2.34 (1.46–3.76)**
				male sex: **0.54 (0.35–0.83)**
				wound score: **0.72 (0.53–0.97)**
Model 4				
Post-procedural eABPI <0.9 (reference)	84	44 (67%)	1	1
Post-procedural eABPI ≥0.9	90	64 (94%)	**1.7 (1.2–2.6)**	**2.05 (1.34–3.14)**
				male sex: **0.54 (0.35–0.83)**
				wound score: **0.72 (0.53–0.97)**

eABPI = estimated ankle-brachial pressure index, baseline eABPI is pre-procedural eABPI.

### Sensitivity analyses

We performed additional KM sensitivity analyses including all patients (*n* = 246) according to baseline PAD status of patients (claudication *vs* CLTI and Fontaine stage), and quartiles of pre-procedural eABPI (Supplementary Table 1). Likely due to the small number of patients with claudication (*n* = 20) and low event rates in this group, there were overall no significant differences in outcomes between patients with claudication and CLTI at baseline (statistical power <50%). There were, however, significantly higher rates of mortality and major amputation contributing to lower AFS and higher MALE between Fontaine stages. Note that there were no 1-year major amputations, and no cdTLR or MALE in patients with claudication. None of the 1-year outcomes (wound healing, mortality, amputation, cdTLR, AFS, MALE) differed between quartiles of pre-procedural eABPI in all patients (*n* = 246) and when restricted to those with CLTI (*n* = 226, Supplementary Tables 1D and 2D). Supplementary Table 2 shows analyses comparing outcomes in patients with CLTI with only pre-procedural eABPI *vs* those with both pre- and post-procedural eABPI; and comparing outcomes after procedures that were technically successful *vs* those that were not. This showed that both failure to achieve technical success and missing post-procedural eABPI were associated with higher mortality and lower AFS. This may be explained by lower frequencies of technical success in patients missing post-procedural eABPI. Finally, we performed survival analyses with all patients including claudication and CLTI according to post-procedural eABPI quartiles (all *n* = 219). The results were similar to the main analyses restricted to CLTI.

## Discussion

In the current study, ABPI was estimated by ankle ultrasound (eABPI) as previously described,^13^ and it is the first study to investigate post-procedural eABPI as a predictive biomarker of outcome after angioplasty. We have demonstrated that improved eABPI early after interventions (1 month) predicts favourable 1-year clinical outcomes and may help to answer the question as to what level of perfusion is required to achieve optimal clinical outcome in patients with CLTI. Our data show that all clinic outcomes, namely wound healing, survival, AFS, freedom from MALE, cdTLR, and major amputation were significantly better in patients with ≥0.7. All outcomes except for survival remained significant after adjustment for baseline eABPI, age, sex, number of-comorbidities, number of intervened segments, WIfI wound score, and WIfI foot infection scores where age, co-morbidities, and foot infection were the major significant drivers of survival. Importantly, our data indicate that ≥0.7 may not be enough because post-procedural eABPI of <0.9 was still associated with lower wound healing and higher MALE and cdTLR as compared to ≥0.9 and this remained significant after adjustment for confounding variables.

Previous studies have shown that an ABPI of ≤0.90 is independently and robustly associated with increased risk of severe ischaemic leg outcomes, supporting ABPI ≤0.90 as a threshold for diagnosing PAD.^16^ A meta-analysis also indicated that a low ABPI is associated with an increased risk of subsequent cardiovascular and cerebrovascular morbidity and mortality.^17^ Our sensitivity analysis did not show that pre-procedural eABPI predicted the outcome. This contrasts with a previous study that reported pre-procedural ABPI values of <0.5 were a strong predictor of adverse outcome after angioplasty.^18^ In that study,^18^ post-procedural values were not measured making it hard to compare with our work. Future studies need to address whether an early angioplasty eABPI result of <0.7 or <0.9 can be accepted, or should trigger further interventions in particular if wounds appear clinically static. The next step could be the implementation of ‘on table’ perfusion monitoring, e.g., with eABPI or similar approach,^19^ aiming at perfusion target values of >0.7 or >0.9 for revascularisation and taking the qualitative ‘straight-line flow to the foot’ paradigm to the next logical and quantitative level.^20^ This needs to be addressed in future studies.

Whereas current clinical PAD guidelines cautiously recommend that ABPI should be measured after endovascular revascularisation as part of the surveillance program during a follow-up visit together with a clinic exam, no studies to date have directly investigated the value of ABPI after endovascular revascularisation to predict the clinical outcome.^3^^,^^5^^,^^11^^,^^12^ We were not able to identify a single study that has evaluated the prognostic value of the (e)ABPI value achieved after angioplasty. However, the validity of our approach, based on Doppler wave form characteristics, is supported by recent work of others showing that post-procedural acceleration time in pedal or distal crural arteries can predict limb salvage and wound healing.^19^^,^^21^ Of note, our approach to estimate eABPI is based on an empirical non-linear relationship between acceleration index and ABPI in patients without media sclerosis.^13^ In the previous paper,^13^ we had also performed regression analyses to find the best–fit curve to estimate ABPI not only testing the acceleration index, but also evaluated peak velocity and acceleration time. We had chosen acceleration index over acceleration time and peak velocity because it was the strongest predictor of eABPI and performed better than the other characteristics. The major advantage of the mathematical transformation of acceleration index into ABPI-units and reporting as eABPI is that the units are more accessible to clinicians and can be directly integrated into current practise as an alternative or even replacement for standard ABPI as it is independent of media sclerosis. To improve dissemination and implementation of our results, the mathematical transformation of acceleration index into eABPI could be simply integrated into automatically calculated functions in the ultrasound machine's onboard software. While this is also theoretically possible with acceleration time, the accuracy to predict ABPI is likely lower based on our previous work.^13^ The advantage of the pedal acceleration time over our current ankle based eABPI approach is that it also assesses perfusion further distal below the ankle^22^ and may pick up remaining foot lesions and not only leg ischaemia potentially similar to toe brachial index.^23^

As this is a single-centre observational study, further external validation studies are required to ensure generalisability of results. We have recently validated the ankle-Doppler based eABPI approach^13^ and it is now routinely used for ABPI assessment in our institutions. As standard ABPI was not measured in the current study, we cannot compare the performance of eABPI to predict clinical outcome with standard ankle compression ABPI. Although the majority of patients received eABPI measurements after endovascular procedures, some data were missing (11%). This is unlikely to have affected the results of our analyses in a major way. The patients with missing data had low eABPI at baseline, a large proportion of non-successful procedures, and overall worse outcome than patients with complete data. Furthermore, the numbers of patients with claudication (*n* = 20) and events in claudicants (*n* = 2 deaths) were too low to assess the prognostic value of eABPI after interventions in this group. Therefore, we focused the main analyses and conclusions of the current study on CLTI. The assessment of wound healing was based on electronic notes of podiatry and vascular clinic appointments. Therefore, the true timepoint of wound closure may slightly differ from the reported results. Besides ABPI, other factors like inflammation and wound size may influence the outcome in CLTI. At the time of prospective data gathering, we did not have data available on limb threat severity (e.g., WIfI score) as they were not routinely documented in the electronic patient records during the time of the study. However, we were able to retrospectively gather wound and foot infection scores. As this component of the analysis was retrospective it may carry some uncertainty. Future studies need to take this into account. Finally, the study results cannot address one of the most important questions, if (e)ABPI is superior to clinical monitoring alone. We believe that clinical monitoring may be sufficient when patients with CLTI present with clear ischaemic rest pain or present with typical claudication. However, patients with CLTI and tissue lesions are often more difficult to evaluate and the expected ischaemic tissue healing time is long, even after successful revascularisation (overall median healing time 168 days). However, failure to improve or recurrent symptoms surely require repeated assessment. Whether repeated eABPI measurements beyond the early phase after intervention - with triggered secondary procedures - are effective to improve outcome of patients, needs to be shown.

In conclusion, this study demonstrates that post-procedural eABPI provides clinically important prognostic and predictive information. Our data indicate that revascularisations should target values of at least 0.9 to achieve optimal outcomes. We highlight the importance of post-procedural follow-up with haemodynamic assessment and suggest that eABPI could become integral as an efficient haemodynamic prognostic and predictive biomarker for assessment of clinical outcome, independent of media sclerosis.

## Contributors

Conceptualization, ADR and CH; Data curation, CH; Formal analysis, JH, SSS and CH; Investigation, CA, FPC, RJ, NN, IW, RB, AP, and CH; Methodology, JH, LH, MBW, SSS, RHJ and CH; Project administration, CH; Resources, CH; Software, CH; Supervision, AR, GDM, RJ and CH; Validation, ADR, JH, LH, CA, FPC, MBW, RB, SSS, BCTF, GDM and CH; Visualization, CH; Writing – original draft, CH; Writing – review & editing, ADR, JH, LH, CH, RJ, FPC, MBW, NN, IW, RB, SSS, AP, BCTF, GDM and CH. CH, SSS, JH, LH, ADR had access to and verify the underlying study data.

## Data sharing statement

Data will not be shared as this is a service evaluation. Further enquiries could be sent to the corresponding author.

## Declaration of interests

CH has received honoraria for presentations by Bayer and is member of the board of *European Society of Vascular Medicine* and board of the *European Society of Cardiology Working Group on Aorta and Peripheral Vascular* *Disease* and member of the *Royal Society of Medicine Vascular, Lipid and Metabolic Medicine Council*. All other authors declare no competing interests.
