# Supplementary material for: Prognostic and predictive value of ultrasound-based estimated ankle brachial pressure index at early follow-up after endovascular revascularization of chronic limb-threatening ischaemia: a prospective, single-centre, service evaluation
Source: eClinicalMedicine. 2024 Jan 5;68:102410. doi: 10.1016/j.eclinm.2023.102410 (PMC10809069; doi:10.1016/j.eclinm.2023.102410)
Supplement: Supplementary data [file mmc1.docx]

**SUPPLEMENTARY DATA**

**Supplementary TABLE 1:** **Sensitivity analysis.** Kaplan Meier survival analysis comparing baseline pre-procedural characteristics; (A) baseline PAD status (intermittent claudication [CI] vs chronic limb-threatening ischaemia [CLTI]), (B) Fontaine stages, (C) quartiles (Q1-4) of baseline estimated ankle brachial pressure index (eABPI). (cdTLR=clinically driven target lesion revascularisation, MALE=major adverse limb event [cdTLR, major amputation], total *n* are number at risk, mean time to events in days).

**Supplementary TABLE 2:** **Sensitivity analysis.** Kaplan Meier survival analysis patients with chronic limb-threatening ischaemia (CLTI) comparing (A) patients that had only pre-procedural eABPI and those with both pre- and post-procedural eABPI measurements, (B) patients with and without technically successful procedures and (C) quartiles of pre-procedural eABPI. (cdTLR=clinically driven target lesion revascularisation, MALE=major adverse limb event [cdTLR, major amputation], total *n* are number at risk, mean time to events in days).

**Supplementary TABLE 3:** **Sensitivity analysis.** Kaplan Meier survival analysis in entire cohort including both patients with intermittent claudicantion (CI) and chronic limb-threatening ischaemia (CLTI) comparing quartiles of post-procedural eABPI. (cdTLR=clinically driven target lesion revascularisation, MALE=major adverse limb event [cdTLR, major amputation], total *n* are number at risk, mean time to events in days).
